# Assessing association of preoperative estimated glomerular filtration rate with postoperative acute kidney injury in very elderly surgical patients: ***Re: Wu Q, et al.* Predictive role of estimated glomerular filtration rate prior to surgery in postsurgical acute kidney injury among very elderly patients: a retrospective cohort study. Ren Fail. 2019;41(1):866–874.**

**DOI:** 10.1080/0886022X.2020.1760886

**Published:** 2020-05-02

**Authors:** Lei Wan, Fu-Shan Xue, Shao-Hua Liu, Peng Dong

**Affiliations:** Department of Anesthesiology, Beijing Friendship Hospital, Capital Medical University, Beijing, People’s Republic of China

Sir,

By a retrospective cohort study, Wu and colleagues [1] assessed predictive role of preoperative estimated glomerular filtration rate (eGFR) for postoperative acute kidney injury (AKI) in very elderly surgical patients and showed that eGFR could be used as an index for AKI risk stratification. This study has potential clinical implications, but we noted several issues that wished to remind readers for attention and obtain the authors’ comments.

First, other than preoperative health and comorbidities, surgical burden is an important determinant for the development of postoperative AKI [2]. The authors provided types of surgery, but these data could not adequately reflect the surgical risk, which has been significantly associated with the development of postoperative AKI in elderly surgical patients [3]. There are many recognized surgical risk scoring systems available in clinical practice, such as the National Surgical Quality Improvement Program Risk (NSQIP) for serious complications, the NSQIP for surgical risk calculator, the Physiological and Operative Severity Score for the enUmeration of Mortality and Morbidity (POSSUM) and the Preoperative Score to Predict Postoperative Mortality (POSPOM), surgical risk score, surgical Apgar score and others. We believe that findings of this study would be more informative, if the design had taken a surgical risk score as potential risk factor of AKI.

Second, this study observed the AKI occurred within 7 days after surgery. The readers were provided with postoperative contract exposure, blood transfusion and outcomes, but not the occurrence of postoperative adverse events and complications. It must be noted that postoperative AKI is not a single disease but rather a syndrome comprising multiple clinical conditions, i.e., the development of postoperative AKI is the consequence of complex interactions among many perioperative predisposing and precipitating factors. The available evidence indicates that postoperative cardiogenic shock, hemodynamic instability, anemia, hypovolemia or fluid overload, blood transfusion, infection, sepsis, exposure to vasopressors and diuretics, and use of nephrotoxic drugs are common precipitating factors for AKI [4–6]. In fact, early postoperative AKI occurred within 48 h following surgery is mainly associated with preoperative comorbidities and intraoperative factors, whereas late postoperative AKI is closely associated with postoperative factors [6]. Thus, we argue that no inclusion of postoperative risk factors in the model would have tampered with inferences of multivariable analysis when determining risk factors of postoperative AKI and their adjusted odds ratios.

Third, the authors provided the results of multivariable analysis regarding associations of preoperative eGFR and other covariates with postoperative AKI, but not the results of using univariable analysis to examine multicollinearity among candidate independent variables. Thus, it was unclear why only three perioperative variables were included in multivariable model. Given the specific interests in the roles of some factors for the risk of interested adverse outcome, moreover, it is actually required that all known risk factors associated with the occurrence of interested adverse outcome (including significant and nonsignificant variables in the univariable analysis) should be taken into the multivariable model for adjustment of confounders [7]. Because there was the lack of the data regarding univariable analysis, we were concerned that some risk factors associated with the development of postoperative AKI, such as intraoperative hypotension and postoperative blood transfusion, had been missed.

Finally, when determining the effect of AKI on postoperative death by the Cox proportional hazards regression analysis, it was also unclear why three models were established for separate adjustment of age, gender and APACHE II score. In fact, all known factors affecting postoperative death should be taken into the multivariable model for statistical adjustment. After multivariable analysis, independent contributions of significant factors including AKI development and its stages to patients’ outcomes can be obtained according to their odds ratios and 95%, confidence intervals and *P* values. Furthermore, this study only focused the effects of AKI occurrence and its severity on postoperative death, but not the influence of AKI duration. It must be emphasized that duration of AKI, especially for persistent AKI, is more important factors affecting postoperative short- and long-term adverse outcomes of patients. The longer postoperative AKI is, the more frequent adverse outcomes occur [2]. Furthermore, it has been shown that combination of AKI duration and severity is better in predicting postoperative mortality than AKI severity alone [8].
